# Epigenetic clocks and research implications of the lack of data on whom they have been developed: a review of reported and missing sociodemographic characteristics

**DOI:** 10.1093/eep/dvad005

**Published:** 2023-07-15

**Authors:** Sarah Holmes Watkins, Christian Testa, Jarvis T Chen, Immaculata De Vivo, Andrew J Simpkin, Kate Tilling, Ana V Diez Roux, George Davey Smith, Pamela D Waterman, Matthew Suderman, Caroline Relton, Nancy Krieger

**Affiliations:** Population Health Sciences, Bristol Medical School, University of Bristol, Bristol BS8 2BN, UK; Integrative Epidemiology Unit, Population Health Sciences, Bristol Medical School, University of Bristol, Bristol BS8 2BN, UK; Department of Social and Behavioral Sciences, Harvard T.H. Chan School of Public Health, Harvard University, Boston, MA 02115, USA; Department of Social and Behavioral Sciences, Harvard T.H. Chan School of Public Health, Harvard University, Boston, MA 02115, USA; Program in Genetic Epidemiology and Statistical Genetics, Department of Epidemiology, Harvard T.H. Chan School of Public Health, Boston, MA 02115, USA; Department of Medicine, Harvard Medical School, Brigham and Women’s Hospital, Boston, MA 02115, USA; School of Medicine, National University of Ireland Galway, Galway H91 TK33, Ireland; Population Health Sciences, Bristol Medical School, University of Bristol, Bristol BS8 2BN, UK; Integrative Epidemiology Unit, Population Health Sciences, Bristol Medical School, University of Bristol, Bristol BS8 2BN, UK; Department of Epidemiology and Biostatistics and Urban Health Collaborative, Dornsife School of Public Health, Drexel University, Philadelphia, PA 19104, USA; Population Health Sciences, Bristol Medical School, University of Bristol, Bristol BS8 2BN, UK; Integrative Epidemiology Unit, Population Health Sciences, Bristol Medical School, University of Bristol, Bristol BS8 2BN, UK; Department of Social and Behavioral Sciences, Harvard T.H. Chan School of Public Health, Harvard University, Boston, MA 02115, USA; Population Health Sciences, Bristol Medical School, University of Bristol, Bristol BS8 2BN, UK; Integrative Epidemiology Unit, Population Health Sciences, Bristol Medical School, University of Bristol, Bristol BS8 2BN, UK; Population Health Sciences, Bristol Medical School, University of Bristol, Bristol BS8 2BN, UK; Integrative Epidemiology Unit, Population Health Sciences, Bristol Medical School, University of Bristol, Bristol BS8 2BN, UK; Department of Social and Behavioral Sciences, Harvard T.H. Chan School of Public Health, Harvard University, Boston, MA 02115, USA

**Keywords:** DNA methylation, epigenetic age, health equity, socioeconomic

## Abstract

Epigenetic clocks are increasingly being used as a tool to assess the impact of a wide variety of phenotypes and exposures on healthy ageing, with a recent focus on social determinants of health. However, little attention has been paid to the sociodemographic characteristics of participants on whom these clocks have been based. Participant characteristics are important because sociodemographic and socioeconomic factors are known to be associated with both DNA methylation variation and healthy ageing. It is also well known that machine learning algorithms have the potential to exacerbate health inequities through the use of unrepresentative samples – prediction models may underperform in social groups that were poorly represented in the training data used to construct the model. To address this gap in the literature, we conducted a review of the sociodemographic characteristics of the participants whose data were used to construct 13 commonly used epigenetic clocks. We found that although some of the epigenetic clocks were created utilizing data provided by individuals from different ages, sexes/genders, and racialized groups, sociodemographic characteristics are generally poorly reported. Reported information is limited by inadequate conceptualization of the social dimensions and exposure implications of gender and racialized inequality, and socioeconomic data are infrequently reported. It is important for future work to ensure clear reporting of tangible data on the sociodemographic and socioeconomic characteristics of all the participants in the study to ensure that other researchers can make informed judgements about the appropriateness of the model for their study population.

## Introduction

DNA methylation (DNAm) is an epigenetic modification to DNA that is involved in a number of aspects of genome regulation. DNAm is dynamic, changing as we age [1], in the course of disease [2] and in the presence of environmental exposures such as smoking [3] and childhood adversity [4, 5]. Ageing is a particularly well-established influence on DNAm, with changes occurring at many DNAm sites across the genome as humans get older [6]. These changes are so reliable that numerous research groups have developed ‘epigenetic clocks’, mathematical models that use DNAm measurements to predict chronological age and other age-related characteristics [7].

The first generation of epigenetic clock methods (e.g. Horvath and Hannum [1, 8]) aimed simply to predict chronological age as accurately as possible. It was observed early on that errors in age prediction, subsequently known as ‘age acceleration’ or ‘age deceleration’ (depending on the direction of the error), were associated with a variety of exposures and diseases as well as mortality risk [9]. These associations suggested that, beyond chronological ageing, these clocks may actually provide a measure of biological ageing (which refers to the progressive decline of the body’s physiological functions [10]). To enhance this feature, a second generation of epigenetic clocks was developed; these incorporated health biomarkers and phenotypes alongside chronological age in the model. Increasing numbers of studies are now considering how social determinants of health might impact epigenetic ageing, with evidence indicating positive associations between accelerated epigenetic aging and social adversity measured in relation to socioeconomic position [11], education [12], neighbourhood characteristics [13–15], and racial discrimination [15].

Although the epigenetic clocks themselves have been quite extensively reviewed [7, 16–18], relatively little attention has been paid – in both these review articles and also the empirical studies that use these clocks – to the sociodemographic characteristics of the individuals whose data have been used to develop these clocks. This sociodemographic information is important in order to assess the generalizability of the clocks to different populations, given robust evidence that (i) variation in DNAm is altered by genetic, social, and environmental factors [3, 19–24], (ii) adverse social and biophysical exposures are socially patterned and affect the risk of disease and also processes of healthy ageing [25–27], and (iii) study selection pressures (which may be related to sociodemographic characteristics) can induce biased study estimates [28–31], which could lead to problems with clock generalizability. Our concern is that for these reasons an algorithm developed to estimate ageing using DNAm in one population may not translate to other populations with divergent sociodemographic or socioeconomic profiles. For example, a clock may miss environmental impacts on the methylome that contribute to ageing in some populations if it is developed in a population with low exposure to those impacts, e.g. pollution or social adversity [32].

This is not a new concern, as there is existing literature describing the potential for machine learning algorithms to exacerbate health inequities through the use of unrepresentative samples – this may happen when prediction models underperform in social groups that were poorly represented in the training data used to construct the model [33–35]. If prediction models underperform, then estimates will be less accurate, which could bias associations in either direction. Because of the nature of epigenetic clocks, the direction of bias is likely to be quite complicated. Epigenetic clocks are made up of many CpG sites (often hundreds) scattered across the genome. The dynamic and regulatory nature of DNAm therefore implies that clocks will integrate biological signals from a wide variety of biological functions and pathways (which for the most part are unknown). The responsive nature of DNAm implies that they will capture variation due to a multitude of endogenous biological factors and external exposures that differ between populations. As such this bias may either over- or under-estimate the effects, and we do not think it would be easy to predict which direction the bias might go, even for specific studies.

The possibility of bias has already been described for PhenoAge, one measure of epigenetic age [36]; it is a particular concern for epigenetic data as two recent reviews show that, at least in regard to racialized groups, epigenetic data are predominantly from individuals of ‘European’ heritage [37, 38] (although no such review has been conducted for other sociodemographic characteristics). Wide-spread inconsistencies in the literature about associations with epigenetic age acceleration support this concern, as inconsistencies are likely driven by differences in characteristics between study populations. For example, inconsistent associations have been reported with education [12, 15, 39–42], socioeconomic status [15, 39, 41, 43–50], and racialized group [51–54]; as well as inconsistent associations between epigenetic age acceleration and health and social outcomes when analyses are stratified by sociodemographic characteristics, such as education level [55], country of birth [40], and racialized group [42, 55, 56]. One recent study reported that when testing the association between epigenetic clocks and healthspan-related characteristics, smaller effect sizes were found for Black American participants in comparison to white American participants [54], suggesting it is possible that associations could be biased towards the null in some study populations. These inconsistencies may be due in part to some clocks including loci known to be differentially methylated by country of birth [40] and racialized group [36, 45].

Whatever the reason for these inconsistencies, it is clear that any interpretation of associations with epigenetic age acceleration should take into account any differences between the populations in which the epigenetic clock was derived and the populations where the associations were observed. To assist in these comparisons, we collate for the first time the basic social characteristics of the participants utilized in the development of 13 commonly used epigenetic clocks.

## Results

Table 1 contains extracted information from each of the clock papers. Supplementary Table S1 reproduces Table 1 along with additional details of the conceptualization of racialized groups and genders. Supplementary Table S2 provides details of how each clock was constructed and the specific datasets used by each paper.

**Table 1:** sociodemographic and socioeconomic characteristics of participants whose data were utilized to create the 13 epigenetic clock papers

Clock	Age (years) and year at time of specimen collection; birth cohort	Racialized groups	Country	Sex or gender	Nativity	Economic measures	Education	Sociodemographic characteristics used in model constructing clock?	Report validation across sociodemographic groups?
Horvath [1]	Age: 0–100; year: not stated in paper; birth cohort: cannot determine	No specification of terminology; the term ‘ethnicity’ is used with regard to one of the studies. Training: reported for 5/39 datasets; one study comprising four datasets includes individuals of ‘non-Hispanic Caucasian ethnicity’ (4 brain regions from the same individuals), and one study includes ‘Taiwanese’ individuals. Test: reported for 1/32 datasets; one study includes ‘Gambian’ individuals	Training: reported for 3/39 datasets – two datasets from one study include data from the Netherlands and one dataset includes data from the USA. Test: reported for 2/32 datasets – two datasets drawn from one study include data from the UK	No specification of terminology; terms ‘male’ and ‘female’ only. Training: 37% female. Test: 8% female	Not stated in paper	Not stated in paper	Not stated in paper	No	No
Hannum [8]	Age: 19–101; year: not stated in paper; birth cohort: cannot determine	Term ‘ethnicity’ with no explicit definition. Training: ‘426 Caucasian and 230 Hispanic individuals’	Not stated in paper	Terms ‘gender’, ‘women’, and ‘men’ with no explicit definition. Training: 52% women	Not stated in paper	Not stated in paper	Not stated in paper	Gender and ethnicity (no definition of either; ethnic groups included are ‘Caucasian and Hispanic’)	No. Compare the ageing rate between men and women in training data; but no test dataset is used so not considered validation
epiTOC [59]	Age: 19–101; year: not stated in paper; birth cohort: cannot determine	Not stated in paper	Not stated in paper	Not stated in paper	Not stated in paper	Not stated in paper	Not stated in paper	Sex (no definition)	No
Zhang’s elastic net [58]	Age: 2–104; year: not stated in paper; birth cohort: reported for two cohorts – one born in 1921 and the other born in 1936	No specification of terminology. Training: reported for one of 14 cohorts; ‘the MND cohort is from a systems genomics study of Motor Neuron Disease in Chinese subjects’	Training: Reported for 4/14 cohorts – three cohorts from Scotland and one from Australia	Not stated in paper	Not stated in paper	Not stated in paper	Not stated in paper	No	No
MiAge [66]	Age: not stated in paper, included only as *Y*-axis on some plots (∼20–90 years old); year: not stated in paper; birth cohort: cannot determine	Not stated in paper	Not stated in paper	Not stated in paper	Not stated in paper	Not stated in paper	Not stated in paper	No	No
DNAmTL [64]	Age – training: 22–93, Test 1: 22–82, and Test 2: 21–100; year – training: stated for 1/2 cohorts: 2000–04, Test 1: stated for 1/3 cohorts: 2000–04, and Test 2: stated for 4/9 cohorts: 1998–2007; birth cohort – training: 1/2 cohorts: 1906–81, Test 1: 1/3 cohorts: 1920–81, and Test 2: 4/9 cohorts: 1915–86	Terms ‘ethnicity’, ‘race/ethnicity’, and ‘ancestry’ used with no definition. Training: 19% ‘European ancestry’ (text)/‘European’ (tables) and 81% ‘African Ancestry’ (text)/‘African American’ (tables). Test 1: 14% ‘African Ancestry’ (text)/‘African American’ (tables), 86% ‘European ancestry’ (text)/‘European’ (tables). Test 2: 77% ‘European ancestry’ (text)/‘European’ (tables), 15% ‘African Ancestry’ (text)/‘African American’ (tables), 8% ‘Hispanic ancestry’ (text)/‘Hispanic’ (tables)	Training: reported for 1/2 – one in the USA. Test 1: reported for 2/3 – two in the USA. Test 2: one in the UK, two in Scotland, one in the USA, one in Italy	Term ‘sex’ used with no definition; terms males/females and men/women used. Training: 75% female. Test 1: 56% female. Test 2: 74% female	Not stated in paper	Not stated in paper	Reported for 5417 of 9875 (54.9%) of test dataset participants: 16.6% less than high school, 16.7% high school degree, 33.6% some college, 33.2% college degree and beyond	No	Yes. Compare the DNAmTL correlation with chronological age between men and women – in Framingham Heart Study (FHS) (test dataset), higher correlation in men (0.39 vs. 0.26) even though training data were 75% women. In Bogalusa Heart Study (BHS) (test dataset), higher correlation in women (0.48 vs. 0.34). Also compare between the racialized groups in one test dataset – in BHS no difference between correlations between ‘black’ and ‘white’ participants (0.36 vs. 0.35)
Bocklandt [60]	Age: 21–55; year: not stated in paper; birth cohort: cannot determine	Not stated in paper	Not stated in paper	Terms ‘male’ and ‘female’ used with no definition. Training: 100% male. Test: 48% female	Not stated in paper	Not stated in paper	Not stated in paper	No	Yes. Compare the correlation between predicted age and chronological age in males and females in test dataset. The predictor was trained in a male-only sample; higher correlation was observed in males (0.83) vs. females (0.75), with a higher error (difference between observed and predicted age) in the females (6.2 vs. 5.3 years for males)
Koch/Wagner [61]	Age: 16–72; year: not stated in paper; birth cohort: cannot determine	Not stated in paper	Not stated in paper	Term ‘women’ only used in reference to one dataset. Training: not reported. Test: reported for 1/8 datasets – 100% ‘women’	Not stated in paper	Not stated in paper	Not stated in paper	No	Yes. State that ‘Gender-related differences in the age-predictions were not observed using this signature (data not shown).’
Weidner [57]	Age: 0–78; year: not stated in paper; birth cohort: cannot determine	Not stated in paper	Training 1 and Test 1: not reported. Training 2: Germany. Test 2: Germany	Term ‘gender’ used, with ‘males’ and ‘females’. Training 1: 81% female. Test 1: unclear. Training 2: unclear. Test 2: unclear	Not stated in paper	Not stated in paper	Not stated in paper	No	Yes. Compare the difference between predicted and chronological age for males and females in test dataset in plot in figure 3, showing no difference at *P* = 0.05, but do not provide statistics or elaborate on comparison. In the Supplementary material, compare the difference between predicted and chronological age for differing numbers of years in education, showing no difference at *P* = 0.05
Zhang’s mortality [62]	Age – training: 50–75, test: 31–82; year – training: 2000–02, test: 2006–08; birth cohort – training: born 1925–50, test: born 1924–75	Not stated in paper	Training: Germany. Test: Germany	Term ‘sex’ used with terms males/females and women/men (males/females in table where % is taken from). Training: 52% female. Test: 50% female	Not stated in paper	Not stated in paper	Not stated in paper	Sex (not defined)	Yes. Downstream association analyses in both training and test stratified by sex/gender. Most associations stronger in women in training data; most associations stronger in men in test data (similar proportion of men and women in training and test datasets)
PhenoAge [63]	Age – biomarker selection: 20+, training: 21–91 (baseline) and 30–100 (follow-up), test: 50–80; year – biomarker selection: 1988–94 and 1999–2014, training: 1998 (baseline) and 2007 (follow-up), test: data presented for 4/5 datasets: 1993–2008; birth cohort – biomarker selection: before 1974–94, training: 1907–77, test: 1913–43	Terms used: ‘race/ethnicity’ and ‘ancestry’, no definition. Biomarker selection: not stated in paper. Training: no data presented. Test: data presented for 4/5 datasets (72% of participants): 18% ‘Black’, 26% ‘African American’, 11% ‘Hispanic’ 41% ‘White’	Biomarker selection: the USA. Training: no data presented. Test: data presented for 4/5 datasets, all based in the USA	No mention for DNAm data other than cohort description and no definition. Biomarker selection: not stated in paper. Training: no data presented. Test: data presented for 2/5 datasets (27% of participants) – 54% ‘men’ and 46% ‘women’	Not stated in paper	Not stated in paper	Not stated in paper	No	Yes, across racilaized groups in the test dataset (Black, white, and Hispanic groups). Test via the correlation between DNAm age and chronological age showed similar correlations for Black and white participants, with a slightly higher correlation for Hispanic participants
DunedinPoAm [65]	Age – biomarker selection: 26, 32, and 38, training: 38, test: reported for 3/4 datasets (81% participants): 18–95; year – biomarker selection: 1998, 2004, and 2010, training: 2010, test: 1999–2013; birth cohort – biomarker selection and training: April 1972–March 1973, test: (3/4 datasets) 1917–95	Terms used: only ‘white’ and ‘of white European descent’. Biomarker selection and training: 93% were ‘white’/‘of white European descent’; not specified for the remaining 7% of participants. Test: ‘mostly of white European descent’; data reported for 1/4 datasets: 77% ‘white’	Biomarker selection and training: New Zealand. Test: reported for 3/4 datasets – two from the UK and one from the USA	Terms ‘sex’, ‘male’/‘men’, and ‘female’/‘women’ used, no definition. Biomarker selection and training: 48% female. Test: reported for 3/4 datasets – 61.5% male	Biomarker selection and training: 100% born in Dunedin, New Zealand. Test: not stated	Biomarker selection and training: ‘The cohort represents the full range of socioeconomic status on NZ’s South Island’. Test: reported for 1/4 datasets (41% of test participants) – 25.6% of E-Risk families lived in ‘wealthy achiever’ neighbourhoods compared to 25.3% nationwide; 5.3% vs. 11.6% lived in ‘urban prosperity’ neighbourhoods; 29.6% vs. 26.9% lived in ‘comfortably off’ neighbourhoods; 13.4% vs. 13.9% lived in ‘moderate means’ neighbourhoods; and 26.1% vs. 20.7% lived in ‘hard-pressed’ neighbourhoods	Not stated in paper	No	Yes. Includes slopes for ‘men’ and ‘women’ for one of the test datasets in figure 3 of the DunedinPoAm paper, but no statistics are reported. Compare clock estimates between participants from low, middle, and high social class categories, with those from the lower socioeconomic groups having faster clock ageing
GrimAge [55]	Age – biomarker selection and training: 59–73, test: 46.5–78; year – biomarker selection and training: not reported, test: reported for 2/5 test datasets: 1998–2007; birth cohort – biomarker selection and training: not reported, test: for 2/5 datasets: 1920–57	Terms used: ‘race/ethnicity’ and ‘ancestry’. Biomarker selection and training: not stated in paper. Test: 50% ‘European ancestry (Caucasians)’, 40% ‘African Americans’, 10% ‘Hispanic ancestry’	Biomarker selection and training: the USA. Test: the USA (2/5) and Italy (1/5)	Terms ‘sex’ and ‘male’ and ‘female’ used, no definition. Biomarker selection and training: 54% female. Test: 83% female	Not stated in paper	Not stated in paper	Stated for 3079 (41.7%) of 7375 test dataset participants: 8.8% less than high school, 14.8% high school degree, 52% some college, 24.2% college degree and beyond	Sex (not defined)	Yes. Stratified test datasets by racialized group membership to test whether the mortality predictors applied to each group. However, do not make direct comparisons in the text. Stratify by educational attainment in a Supplementary analysis and find that AgeAccelGrim remains a significant predictor of time-to-death for all education levels (although the lowest HR for less than high school education); same for time to Coronary Heart Disease. AgeAccelGrim is stratified by racialized group in a Supplementary analysis with differences between groups, with higher SDs in the black participants and the lowest in the Hispanic participants, although this is not discussed

### Age, Biological Specimen Collection Date, and Birth Cohort

Eleven of the 13 papers report the chronological age of all their training and test dataset participants. Of these, three models included children in their training and test datasets: two included participants from birth to adulthood, with no information as to the biological specimen collection dates or birth cohorts of participants (aged 0–100 years [1]; aged 0–78  years [57]), and one model included participants aged 2–104 years old, with 1/14 cohorts born in 1936 and one in 1921 [58]. Four papers reporting the chronological age of all their participants report using only adult participants (aged between 16 and 101 years) without reporting specimen collection date or birth cohort [8, 59–61]. One paper reported using only adult participants (ages 31– 82 years) and reported specimen collection date (2000–08) and birth cohort (1924–75) for all of their participants [62]. One paper reported age for all participants (adults, aged 20–91 years) and reported the specimen collection date (1988–2014) and birth cohort (1907–94) for the majority of their participants (4/5 datasets) [63]. Two papers reported using adult participants (aged 21–93 years), with information on specimen collection date (1998–2007) and birth cohort (1906–86) for some of their participants [55, 64].

One paper reported age for their training data plus 81% of their test data, plus biological specimen collection date for all participants (1998–2013) and birth cohort for the majority of participants (1917–95) [65]. One paper did not report age but included it as the *Y*-axis on plots, illustrating that participants were ∼20–90 years old, with no information on biological specimen collection date or birth cohort [66].

### Sex or Gender

Biological sex or gender were the most frequently reported characteristics, with seven out of 13 clock papers reporting data for all participants, and three out of 13 reporting partial data. Of the seven reporting full data, four report ‘sex’ using the biological sex terms ‘males’ and ‘females’. These papers included between 48% and 83% females in their training and test datasets [55, 62, 64, 65]. One paper reports ‘gender’ using the gendered terms ‘women’ and ‘men’, including 52% women [8], and two report using the terms ‘female’ and ‘male’ without specifying sex or gender, including between 0% and 58% female participants [1, 60]. Of the three studies reporting partial data on sex/gender, one reported on one of eight datasets (100% women, with no use of the terms sex or gender) [61]; one reported on two of five test datasets (46% women, no use of the terms sex or gender) [63]; and one paper reports ‘gender’ using the terms ‘female’ and ‘male’, including 81% female participants in the training data, with no reporting for the test datasets [57]. Three studies use ‘sex’ and one used ‘gender’ as a covariate in the age estimation model, but none conceptualize the reason for this [8, 55, 59, 62].

None of the papers defined what they meant by the terms sex or gender beyond three studies identifying it as a ‘covariate’ or ‘confounder’, so where terminology is mixed it is unclear which specific characteristic is being reported. Being specific about the characteristic that is reported is important because biological sex and gender identity have independent and interacting effects on health [67–70].

### Racialized Groups and Country Context

Only two papers report data on racialized group membership for all the participants in their study, using descriptive terms pertaining to ‘race’, ‘ethnicity’, and ‘ancestry’ (but without defining what these terms mean, whether these data were self-reported by participants or obtained from medical records, and with limited reporting of country context). The paper developing the Hannum clock [8] was developed using a training dataset including ‘426 Caucasian and 230 Hispanic individuals’, with no country context reported. We note that there is scientific recognition that the origins of the term ‘Caucasian’ mean its use should be discontinued – this is because the term was derived from a scientifically fallacious typology that presumed humanity originated in the Caucuses and was ‘white’, with other ‘racial’ groups framed as ‘degenerate’ lineages that branched off of the ‘Caucasian’ trunk [71–73]. The paper developing the DNAmTL clock [64] utilized training and test datasets; the training dataset comprised a total of 2256 individuals from two datasets, of whom 81% were categorized as being ‘African ancestry’ in the text and ‘African American’ in the tables; and 19% as ‘European ancestry’ in the text and ‘European’ in the tables. The country context was provided for one of these datasets (one was based in the US), so it is not possible to fully contextualize the reported racialized groups based on information reported in the paper. Their first test data comprised 1078 individuals from three datasets (Test and Training 1 comprise unique sets of individuals from two datasets). They report that 86% were categorized as being ‘European ancestry’ in the text and ‘European’ in the tables, and 14% as ‘African ancestry’ in the text and ‘African American’ in the tables. The country context was provided for 2/3 of these datasets (two were based in the USA), so again it is not possible to fully contextualize the groups. A second test dataset comprised 9359 individuals from five additional datasets, of whom 77% were categorized as ‘European ancestry’ in the text and ‘European’ in the tables, 15% as ‘African ancestry’ in the text and ‘African American’ in the tables, and 8% as ‘Hispanic ancestry’ in the text and ‘Hispanic’ in the tables. The country context was reported for all five datasets: one in the UK, two in Scotland, one in the USA, and one in Italy.

Five papers that developed an epigenetic clock reported partial data on racialized group membership but did not report clear numbers for all participants in the paper. The paper developing the Horvath clock [1] reported racialized group membership for 5/39 training datasets – participants from four datasets (four brain regions from the same individuals) were categorized as ‘non-Hispanic Caucasian ethnicity’ (country context not reported), and participants from one dataset were categorized as ‘Taiwanese’ (country context not reported). Racialized group membership data was reported for 1/32 test datasets, where participants were categorized as ‘Gambian’ (country context not reported). The paper developing the DunedinPoAm clock [65] reported that 93% of their training dataset participants, all born and residing in New Zealand, were ‘white’ but did not explicitly report data on racialized groups for the remainder of their training data participants. They state that test dataset participants were ‘mostly of white European descent’, reporting that 77% of participants in one of the four test datasets were ‘white’ with no country context; the country context was reported for the other three test datasets with no information on racialized groups (one recruited in the USA and two in the UK). The paper developing the GrimAge clock [55] did not report the racialized group memberships of their US-derived biomarker selection and training dataset (70% of the Framingham Heart Study offspring cohort). Among individuals in their five test datasets, 50% were categorized as ‘European ancestry (Caucasians)’, 40% as ‘African American’, and 10% as ‘Hispanic’; the country context was not integrated, with 2/5 of the test datasets based in the USA and 1/5 in Italy. The paper developing the PhenoAge clock [63] did not report the racialized group membership of the participants in which the biomarkers were selected the National Health and Nutrition Examination Survey III and IV; both are US national datasets), or in whom the DNAm predictor was trained (InChianti, for which they do not report the country context); they reported the racialized group membership of participants in four of the five test datasets, all of which were based in the USA, comprising 72% of test participants, of whom 18% were categorized as being ‘Black’, 26% as ‘African American’, 41% as ‘White’, and 11% as ‘Hispanic’. No data on racialized group membership was provided for the remaining 36% of test participants from two other datasets. Finally, Zhang’s *et al.* paper developing an elastic net predictor [58] reported data on racialized group membership for one of 14 datasets, where they state that the 695 participants from the motor neuron disease (MND) cohort dataset were ‘Chinese’ but did not provide further information or country context.

One study reports only the country context of training and test datasets (both based in Germany) [62]. Another reported only country context for 1/2 training datasets and 1/2 test datasets (both based in Germany) [57]. The remaining four studies [59–61, 66] report no information about the racialized group membership or country context of their participants. None of the papers that did report data categorized by ‘racial’ or ‘ethnic’ terms that they employed explicitly explained or justified their conceptualization or usage of racialized groups, beyond three studies conceptualizing use as a ‘covariate’ or ‘confounder’. One study [8] includes ‘ethnicity’ as a covariate in the age estimation model but does not conceptualize the reasons for this.

### Nativity

Only one study explicitly reported the nativity status of their training dataset participants – 100% of the Dunedin PoAm [65] training dataset study participants were born in New Zealand (with no report as to the nativity status of the test dataset).

### Economic Measures

Only the Dunedin PoAm study [65] included data for some participants on economic status in the paper. For the biomarker selection and training dataset, they state that ‘The cohort represents the full range of socioeconomic status on NZ’s South Island’, but they do not provide descriptive statistics that might enable comparison across datasets. For one of four of their test datasets (41% of test participants), they provide neighbourhood-level socioeconomic data alongside national statistics, illustrating that the sample is broadly representative of the UK in terms of neighbourhood SES.

### Education

Two papers developing epigenetic clocks included data on educational attainment for some of their participants. The first paper, developing the GrimAge clock [55], reported education data for 41.7% of their test dataset participants; they were generally highly educated, with a quarter (24.2%) having a college degree or higher, and only 8.8% having less than high school education (14.8% had a high school degree, and 52% had some college education). The second paper, developing the DNAmTL clock [64], reported education data for 54.9% of their test dataset participants. A higher proportion had less than high school education (16.6%), with the remainder of the sample generally highly educated: 16.7% had a high school degree, 33.6% had some college education, and 33.2% had a college degree or higher. Neither paper reported information on the education level of their training dataset, which is critical to the transportability of the predictor.

### Validation across Social Groups

Eight of the 13 papers reporting epigenetic clock methods report some form of validation of their model in their test data stratified by one or more sociodemographic characteristics. One (developing the DNAmTL clock) compared model performance between men and women in the two test datasets, and additionally between racialized groups in one of these test datasets, by correlating the clock estimate with chronological age [64]. In the first test dataset, they found a lower correlation for women even though the training data comprised 75% women. In the second test dataset, they found a higher correlation for women. We suggest this shows the importance of the need to consider multiple participant characteristics when validating predictive algorithms. There was no difference in clock correlation with chronological age between the two racialized groups (‘black’ and ‘white’ participants). The paper developing Zhang *et al*.’s mortality clock [62] stratified downstream analyses by sex/gender; in their test dataset, they found stronger associations in men despite a slightly higher proportion of women in the training data. The paper reporting the Bocklandt clock [60] used 100% ‘males’ in their training data and tested clock performance in males and females in their test dataset. They found a lower correlation between chronological and predicted age for females compared to males, as well as a higher error between predicted and chronological age for females, suggesting reduced accuracy of the clock. The paper developing the PhenoAge clock [63] looked at the correlation between the age estimator and chronological age stratified by the racialized group – they found slightly higher correlations in ‘Hispanic’ participants, with similar correlations for ‘black’ and ‘white’ participants. The paper developing the GrimAge clock [55] stratified all analyses in the main text by the racialized group, conceptualizing this as testing whether the ageing predictor applied to each group; no conclusions from this were presented in the paper, but in a supplementary analysis shows higher standard deviations of the age estimator for ‘Black’ participants and lower standard deviations for ‘Hispanic’ participants, with ‘white’ participants having standard deviations in the middle of these groups. However, racialized group membership was not reported for the training dataset participants. Additionally, in the supplement they stratify model predictions by educational attainment and find that the model performed for participants of all education levels (although the lowest hazard ratio was found for less than high school education). One paper compared the difference between predicted and chronological age for participants with differing numbers of years in education, showing no difference at *P* = 0.05 [57] and presented plots comparing clock performance in ‘males’ and ‘females’ but did not provide statistics or elaborate on the comparison in the text. One paper developing an epigenetic clock stated that no difference was observed in clock performance between genders but did not present data [61]. One paper presented plots comparing clock performance in ‘males/men’ and ‘females/women’ but did not provide statistics or elaborate on the comparison in the text [65]. One paper compared the ageing rate between men and women but only in the test dataset (which we did not consider to be validation) [8].

## Discussion

The basic sociodemographic characteristics of participants are generally poorly reported in the 13 papers which developed the most popular epigenetic clocks. This makes it challenging for researchers to judge whether the clock is likely to accurately transport to the population they want to study, where the estimation of epigenetic age may be inaccurate in populations with different characteristics, introducing uncertainty to the relationship between epigenetic age and health and social outcomes, therefore biasing estimates in uncertain directions. This is important because different populations are likely to have different socially patterned social, economic, and biophysical exposures that affect their methylomes, and so clocks developed in a socially homogeneous population may not transport well to a population with different social characteristics and different exposures and experiences.

Chronological age was reported for all study participants in 11 of 13 papers. Of these, three include infants and children in model development, meaning that researchers should be cautious about the application of other models to data from children. The majority of other studies included a wide range of adult ages. However, only six of the papers report the biological specimen collection dates, some with information reported on birth cohort (although birth cohort can be derived easily from age and specimen collection date). Biological specimen collection date and birth cohort are important because this means researchers can ascertain whether participants lived through events or situations that might have had impacts on health and health equity. To properly analyse issues related to health equity, it is crucial to combine this with data on place and other sociodemographic characteristics. Participants in the six epigenetic clock models where birth cohort could be derived were born from as long ago as 1906 to as recent as 1995, meaning a range of historical and social events (such as war, economic crisis, and changes in social environment) may have been experienced by participants, depending on their other characteristics.

Where gender or sex was reported, with one exception clocks were trained on both male and female/women and men participants; however, gender or sex was not reported for all participants in five of the 13 studies, and none included any discussion of differences between the influences of sex-related biology and societal gender, or their potential interaction. The two clocks reporting data on the racialized group membership of all participants in their training dataset did not provide country context for all of their participants. Each included individuals from two racialized groups: one included participants predominantly categorized as being ‘Caucasian’ (with no country context) and the other included participants predominantly categorized as being of ‘African ancestry’ or ‘African American’ (with at least some of these participants living in the USA). Only two studies (the papers developing DNAmTL and GrimAge) reported the racialized group membership of all participants in their test dataset (this information is not as pertinent as the training dataset); neither fully integrated country context, with one study including persons located in the USA, the UK, Scotland, and Italy, as well as some participants from unspecified countries, who were categorized as being ‘European’ or ‘European ancestry’, ‘African American’ or ‘African ancestry’, and ‘Hispanic’ or ‘Hispanic ancestry’; the other study included persons living in the USA and Italy, as well as unspecified countries, who were categorized as being ‘European ancestry (Caucasians)’, ‘African American’, and ‘Hispanic’. Both of these studies included a majority of participants categorized as being ‘European’ or ‘European ancestry’, some of whom were located in Europe, some in the USA, and for some participants, there was no country context. Only one paper reported the nativity of their participants alongside the country context.

We note that none of the papers that we reviewed explicitly explained or justified their conceptualization or usage of racialized groups, even when they reported data on these characteristics, utilizing them as covariates in the model in one case, or stratified their analysis according to categories they employed for ‘race’, ‘ethnicity’, or ‘ancestry’. This mirrors the findings of a recent systematic review examining how a large number of epigenetic studies poorly incorporate data on social groups and social determinants of health [74]. The inclusion of sociodemographic characteristics as features in models such as epigenetic clocks needs to be thoroughly conceptualized and justified because the inclusion of these characteristics could exacerbate inequities by adjusting away inequalities experienced by individuals with these characteristics [75, 76], meaning inequalities in biological age would be masked by the inclusion in the clock algorithm.

Crucially, only three of the papers we reviewed presented tangible data pertaining to the socioeconomic circumstances of their participants. Two reported education levels for 41.7% and 54.9% of their test dataset participants, where participants were generally highly educated (with the majority having at least some college education). One reported neighbourhood-level economic data for 41% of test dataset participants alongside national figures, illustrating that the sample was broadly representative of the UK. However, none of these papers reported education or economic data for their training data, which are the critical dataset to report. This information is essential to assess the transportability of these clocks to other datasets; it is also important to ensure that health inequalities are not masked or perpetuated in epigenetic research (this may happen when prediction models underperform in social groups that are poorly represented in their training data). The lack of reporting that we find is likely to be due to at least in part the absence of social characteristics in publicly available datasets such as those on GEO; biological data repositories have previously been criticized for a lack of social characteristics of their participants because this prevents the investigation of health inequities that exist between social groups [77]. We would like to reiterate this need for socioeconomic data in the context of epigenetic datasets, as well as the importance of obtaining and reporting these data from cohort studies that have epigenetic data.

Eight of the epigenetic clock models make efforts to validate their models in participants stratified by one or more sociodemographic characteristics, including sex/gender, racialized groups, and education level. Some suggested that there may be little difference between the groups tested, whereas some suggested lower accuracy in groups dissimilar in some way to the training population. However, we suggest that validation methods ought to be improved beyond simple testing of correlation between the clock model and chronological age, or testing downstream associations and that papers should consider multiple sociodemographic characteristics in these validation analyses and ensure to give them due consideration as an important part of the manuscript. None of the eight papers followed up any differences they found in the discussion, or relate differences they find to the characteristics of their training dataset, missing important opportunities to delve into whom these clocks may and may not apply to.

In conclusion, we find that although some of the epigenetic clocks were created utilizing data from datasets including individuals from different sexes/genders and racialized groups, this information is limited by inadequate conceptualization of the social dimensions and exposure implications of gender and racialized inequality, the absence of any socioeconomic data, or any consideration of interactive effects involving these social groups, along with a frequent failure to be clear on the countries from which the data were obtained and also the nativity of the participants. As a result, it is difficult to conclude how transportable the epigenetic clocks with poorly characterized sociodemographic data may be and which social groups they might apply to. Future epigenetic research should ensure to report these important participant characteristics, in combination, to contextualize their work; to properly investigate health inequities, we recommend that at a minimum researchers should collect and report both individual-level and structural-level data as one of our authors has previously suggested [78]. Researchers working with existing methods should ensure they check (where possible) the characteristics of the participants used to generate the clocks against their own population of study. They should also be mindful of the possibility of inaccurate prediction if the population the clock was developed in does differ (or is unknown), and ensure to report this as part of any published work. With the increasing use of epigenetic clocks to conduct work into social determinants of health, an important piece of future work would be to obtain primary data study (where available) to ascertain a more complete picture of the populations in which these epigenetic clocks were developed and what impacts this may have had on the conclusions of subsequent studies using the clocks. This is particularly important to enable studies to address inequalities in health.

## Methods

We included the 13 epigenetic clocks discussed in a recent review that either provide the CpG sites used to construct the clock or provide the means to calculate it [7] – this includes all clocks commonly used in the literature. We extracted participant sociodemographic and socioeconomic characteristics, as reported in the original clock papers and all associated Supplementary material. Where applicable, we extracted information separately for training and test data, as the DNAm data in which the clocks were trained are the most pertinent information. For the second-generation clocks, if biomarkers were selected in a separate cohort, we also extracted information about that cohort. We extracted a number of social characteristics of participants that are important for understanding inequalities in health. The participant characteristics we extracted, as characterized in the studies, pertained to age, biological specimen collection date, and birth cohort; sex or gender; racialized groups (including ‘race’, ‘ethnicity’, and ‘ancestry’); nativity (whether an individual was born in the country of recruitment); country context (the country in which the participants were recruited, identifying the societal structures in which people live); socioeconomic position (e.g. as measured by income, social class); and education; as well as reported validation across social groups. Extracting the data, we use the terms used in the original paper and note where terminology is problematic. Where age and either specimen collection date or birth cohort were reported, we calculated the missing value using the available data.

## Supplementary Material

dvad005_Supp

## Data Availability

All data used in this manuscript are available in the original papers that developed the 13 epigenetic clock methods (all references are contained in Supplementary Table S2).
